# Experiencing stigmatization during the COVID-19 pandemic: a qualitative study among healthcare workers

**DOI:** 10.1186/s12879-025-11486-1

**Published:** 2025-09-09

**Authors:** Dafina Danqa, Marlene Muehlmann, Jule Menzinger, Samuel Tomczyk

**Affiliations:** Department Health and Prevention, Institute of Psychology, University of Greifswald, Robert-Blum-Str. 13, Greifswald, 17489 Germany

**Keywords:** Stigma, Health personnel, Nurses, Physicians, Coping strategies, Qualitative research, Intersectionality

## Abstract

**Background:**

Healthcare workers (HCWs) played a crucial role in dealing with the COVID-19 pandemic. In addition to increased workloads, they were confronted with stigmatization due to their work in the health sector.

**Methods:**

Guided by the Health Stigma and Discrimination Framework (HSDF), this study aimed to explore the experiences of stigmatization of HCWs in Germany using semi-structured interviews (*N* = 34) and investigate effective coping strategies and existing needs in this context. An intersectional perspective was adopted to examine the relevance of multiple stigmatization. The data was analyzed using qualitative content analysis.

**Results:**

The results indicated that HCWs were affected by multiple forms of stigmata (public, anticipated, and self-stigma) from different sources (e.g., in private, public, and work contexts). Different drivers for stigmatization were mentioned, but the fear of infection and its consequences were central. The consequences of the stigmatization experience had a primarily restrictive effect on quality of life and social participation on a psychological, social, and professional level. To cope with the stigmatization, those affected reported behavioral and cognitive coping strategies. In addition to the stigmatization in the context of the COVID-19 pandemic, it became apparent that HCWs experienced additional stigmatization primarily due to their (female) gender, but also due to their profession (i.e., nursing) and (young) age.

**Conclusion:**

Implications for research, policy, and practice were derived from the results. Future research on the topic should also adopt an intersectional perspective in order to be able to depict the complexity of stigmatization processes adequately.

**Supplementary Information:**

The online version contains supplementary material available at 10.1186/s12879-025-11486-1.

In March 2020, the World Health Organization declared a pandemic due to the global spread of the coronavirus (SARS-CoV-2) and the associated disease called COVID-19 [1]. High levels of uncertainty about the new pathogen and the virus’ global emergence led to negative experiences and fears among the global population [2]. Healthcare workers (HCWs) experienced an additional burden through an increased workload and in some cases having to work without appropriate protective clothing posing a heightened risk of infection [3, 4]. As a result of the pandemic, HCWs have had to deal with increased experiences of depression, anxiety disorders, stress, sleep disturbances, and indirect traumatization [5–8]. This may have negatively affected attention and decision-making skills required to deal with the crisis [9]. In addition to multiple challenges posed by the pandemic, HCWs faced the burden of stigmatization; around 30% of HCWs experienced or anticipated infection-related public stigma [10]. HCWs were significantly more likely to experience COVID-19-related stigma compared to individuals not working in this setting [11, 12].

## Stigmatization and infectious diseases

The process of stigmatization includes (a) labelling individuals with certain attributes (e.g., being ill), (b) associating stereotypes and prejudices with those labels (e.g., ill people are dangerous) and (c) categorizing labelled individuals into specific categories, facilitating the formation of 'us' vs. 'them' (e.g., healthy people vs. infected) [13]. In consequence there is a loss of status and an increase in discrimination (e.g., exclusion of people with symptoms of the illness). Stigmatization can only occur when social power differentials are present (e.g., by socially defining what constitutes a negative characteristic). Additionally, different stigmatizing processes can overlap and interact (i.e., double stigmata) resulting in additive or cumulative effects that lead to poorer health outcomes [14].

To reflect the complex nature of stigma and its impact on HCWs during the COVID-19 pandemic, this study applies the Health Stigma and Discrimination Framework (HSDF) [15]. It has previously been applied to the COVID-19 pandemic by Ransing et al. [16] and is suitable for analyzing the stigmatization experienced by HCWs [16–20]. The HSDF divides the process of health-related stigmatization into different domains: (a) individual drivers, (b) reinforcing environmental facilitators, (c) intersectional aspects and double stigma, (d) manifestation of stigmatization, (e) outcomes of stigmatization for affected populations and institutions, and (f) social and health-related impacts [15, 16].

Based on previous research, the main assumed *drivers* of stigmatization of HCWs are fear of contraction in private contexts and the absence of HCWs from work due to illness [17]. Factors *facilitating* stigma include existing social inequities (e.g., xenophobia and racism [21]), lack of regulation (e.g., inadequate anti-discrimination efforts), certain legislation (e.g., mandatory vaccinations for HCWs), and the excessive spread of misinformation (so-called infodemic [16]). For HCWs, facilitators included the lack of adequate transmission control policies, rapidly changing safety standards, and wearing recognizable protective equipment in public, such as scrubs [22]. Sources of stigmatization during the pandemic included friends, acquaintances, family members, superiors, colleagues without contact to COVID-19 patients, patients themselves and their relatives, strangers, and the media [17, 19, 23]. The HSDF suggests adopting an *intersectional perspective*, considering an interplay of sociodemographic factors, health conditions, and professional settings [16]. As for HCWs, there is some evidence, that female HCWs reported stigma more frequently than males [24, 25]. Moreover, the evidence on stigma related to different occupations (e.g., doctors vs. nurses) is inconsistent [25, 26]. *Manifestations* of stigma comprise experiences such as denial of housing, verbal abuse or social devaluation towards HCWs during the pandemic [16], self-stigmatization in terms of self-blame and self-isolation as well as associative stigma towards HCW’s families [17–20]. HCWs themselves also reported stigmatizing attitudes related to COVID-19, e.g., prejudice against people from China by associating them with the virus [27].

As an *outcome*, stigma can result in reduced access to healthcare, reluctance to seek (psychological) help, delayed treatment, and increased infection and death rates in those affected [9, 28–30]. HCWs reported feeling overwhelmed due to stigmatization during the pandemic [31]. This can lead to feelings of isolation, sadness and anxiety, (self-)blame as well as avoidance and distancing or a lack of support and understanding from those closest to them [17, 20, 23]. Experiences of stigmatization further lead to paradoxical emotions in HCWs, such as psychological distress while simultaneously receiving positive reactions from others, like expressions of gratitude, and rejection by other members of society [32]. On an organizational level, stigma can affect the availability and quality of healthcare services, reduce the willingness of HCWs to provide COVID-19 services, and delay preventive care for symptomatic individuals [16].

In sum, stigmatization of HCWs has a broad *impact* on social processes and public health. It can reduce quality of life and well-being on an individual level and affect the healthcare system and economy through increased prevalence of mental illnesses and job or income loss [16]. It can also increase infection rates [9]. In the workplace, stigma can lower motivation and lead to concealment of the disease [16].

## Study aims

In addition to examining the manifestation and experience of stigma, it is also important to investigate coping strategies employed by HCWs to address stigma, as this can inform the development of helpful measures in this context. Some studies identified coping strategies such as concealing one's occupation, discussing it in professional and private contexts, seeking encouragement from others, and educating the public with scientifically based information [17, 23]. However, these aspects were not considered in many previous studies. Therefore, to address this gap in the literature, this study utilizes a qualitative approach to explore the following research questions:

1. *How did HCWs in Germany experience stigmatization in the context of the COVID-19 pandemic?*
2. *How did they cope with this experience?*

## Methods

### Study design

Following an exploratory qualitative design, stigma experiences were collected through semi-structured individual interviews with HCWs using an interview guide [33]. Participants completed a questionnaire assessing socio-demographic information before the interview. All study materials are available as Appendices at https://osf.io/dpq3k/?view_only=a24535ed2a644cd99c9624480a855637 in German (original version) and English (translation). For an overview of the Appendices, see Supplementary Material.

### Sample

As participants, we included all healthcare professions related to health, who had any form of patient contact during the COVID-19 pandemic [34], e.g., nurses, physicians, or medical students. Inclusion criteria comprised being of legal age (18 years), having access to the internet or a telephone, and providing consent to participate. Interviews were conducted by DD in German and online or via phone, without any geographical restrictions. The aim was to gather a sample that broadly reflected the composition of HCWs in the medical context, using purposive sampling [35]. Therefore, the sample selection aimed to achieve a balance of demographic characteristics (e.g., gender, professional group, region of residence etc.). The number of interviews conducted was determined by reaching data saturation, defined as the point during data collection and analysis at which no new relevant themes emerged from the interviews [36].

#### Composition of the research team

The research team consisted of three female researchers (DD, JM, MM) with master's degrees in psychology, and a male professor (ST) of health psychology. Additionally, six Bachelor (LM, LR, LW, KM) and Master (IK, SSP) students in psychology (one male and five female) supported the research at various areas. The age of the team members ranged from 20 to 35 years, with three persons having a migration background (MB). All members of the research team had prior contact with healthcare delivery due to their training as psychologists in clinical settings. The researchers who analyzed the data (DD, JM, LM, MM, SSP) were trained in using MAXQDA 2022 [37].

#### Relationship between researchers and participants

Among other methods, recruitment of participants was carried out through the personal networks of the researchers. Some of the participants were therefore already personally known to the researchers. This applies to three participants (P01, P02 and P14) in the sample. However, these were distant acquaintances, and the relationship lacked depth. While this may have influenced the interview dynamic, a comparison of their interviews with the rest of the sample did not reveal noticeable differences in openness, depth, or content. There were no pre-existing relations to the remaining participants.

### Data collection

Data collection took place between February 2023 and January 2024 and was carried out in two recruitment phases to increase diversity in the sample (e.g., in terms of gender and professional background). In the first phase (February to April 2023), participants P01 to P25 were interviewed. A second recruitment phase followed between August 2023 and January 2024, during which participants P26 to P32 were interviewed. Informed consent and socio-demographic information were collected beforehand via *Unipark* (www.unipark.com). Participants further generated a participant code to connect their questionnaire and interview data in pseudonymized form. To minimize social desirability bias, participants were assured of anonymity and encouraged to speak openly prior to the interviews and audio recordings. Interviews were conducted in a neutral and non-judgmental manner to encourage honest responses. The audio recordings were conducted using iPods as recording devices.

### Materials

#### Questionnaire

Socio-demographic data was assessed through an online questionnaire, which has been guided by the PROGRESS-Plus framework to ensure the collection of equity-relevant data [38].The variables assessed included age, gender, federal state, current job title, direct and indirect MB (derived from the MIGBACK and CORIGIN variables of the SOEP study by Liebig et al. [39]), and socioeconomic information, including educational level, professional qualification, employment status, household information, and net household income, to calculate the socioeconomic status (SES; based on Lampert et al. [40] and Müters et al. [41]). The participant code for pseudonymization was generated based on the recommendation of the Ethics Committee of the German Psychological Society.

#### Interview

The interview guide was developed based on the HSDF [15, 16] and a study conducted by Faller et al. [17], who also interviewed German HCWs. The guide was tested and validated by the first author with three HCWs prior to the main study (see Appendix 2 for details).

The final guide explored the stigmatization experiences of HCWs regarding forms, sources, reasons, reinforcing factors, consequences, and personal coping mechanisms related to stigmatization. Organizational responses, double stigma, and their personal stigmatizing beliefs and actions were also examined. Participants who had not experienced personal stigmatization were asked alternative questions about general impressions of stigmatization, protective factors, and their personal stigmatizing beliefs and actions during the COVID-19 pandemic. After each interview, the guide was evaluated for comprehensibility and participants were invited to add any important information not covered.

### Ethics

The study received ethical approval by the Ethics Committee at the University Medicine Greifswald (reference no. BB 166/22). The collected data was pseudonymized and all audio recordings were deleted after transcription. Participation required (written) informed consent in the pre-interview online survey. All interviewees were offered €30 as compensation.

### Data analysis

Descriptive statistics of socio-demographic data were calculated by DD using IBM SPSS Statistics (Version 27). The individual interviews were coded and qualitatively analyzed using MAXQDA 2022 [37]. The first author DD conducted, coded, and analyzed the interviews. The interviews were audio-recorded and the interviewer (DD) took notes during the conversation, that were transferred into an interview protocol. The audio recordings were transcribed by LW (P01 – P04) using the software *f4transkript* or by an external service (P05 – P36), both following transcription rules based on [42]. All transcripts were anonymized by DD, IK, KM and LR based on pre-defined rules.

A structuring content-analytic approach was adopted to analyze the data, using deductive-inductive category formation according to Kuckartz [43]. First, all relevant text passages were marked and annotated by DD, with notes containing initial analytical ideas. Subsequently, the main thematic categories were formed deductively based on the different topics of the interview guide. In the first coding process, the entire material was coded according to these main categories. To ensure reliability and determine intercoder agreement, eight transcripts (approximately 20% of the material) were coded separately by JM and two research assistants (LM and SSP; female and male). The first four transcripts (LM and SSP: P01, P02, P08, P16) were selected, as they contained a wide range of categories, and were used in an initial agreement process. The remaining four transcripts (JM: P20, P25, P28 and P29) were selected randomly and used in a second agreement process. Intercoder agreement was established through consensual coding. Discrepancies were resolved through discussion, and when no agreement could be reached, a supervising person (ST) was consulted to determine the final coding decision. The percentage of agreement between the independent coders (i.e., assigning the same code to a segment with at least 90% overlap) was repeatedly assessed in this process to refine the category system. A category was labeled problematic and was discussed, if its percentage agreement among the coders fell below 80% [44]. In addition, the intercoder agreement was calculated using the chance-corrected coefficient Kappa (κ) following Brennan and Prediger [45]. The value of κ ranges between −1 and + 1 and is interpreted according to Landis and Koch [46], where a value of 0.61 indicates 'substantial' agreement, while a value of 0.81 or higher indicates 'almost perfect' agreement. In subsequent steps, all text passages coded with the same category were collated and subcategories were inductively formed collaboratively by DD, MM and JM. Definitions and examples for these subcategories were established and documented in the codebook by DD. The entire material was re-coded by DD using the final category system. Finally, DD conducted a category-based content analysis, systematically examining main categories and subcategories.

## Results

### Sample

A total of 36 HCWs participated in the study, of which two (P24 and P27) were excluded from the analysis because they had no patient contact at all during the COVID-19 pandemic as part of their work in the health sector. Their roles consisted of administrative or evaluative tasks (e.g., preparing staff schedules or conducting social-medical assessments), and thus they were not directly involved in patient care. This resulted in a final sample of 34 participants included in the analysis. The mean duration of the interviews was 62 min, varying between 20 and 90 min. The age of the participants ranged from 19 to 63 years (*Mdn* = 28.5, IQR = 17.75). Of the participants, 24 identified as female (70.6%) and 10 as male (29.4%). Seventeen persons (50%) were working in Mecklenburg-Western Pomerania, 13 persons (38.2%) in Berlin, two persons (5.9%) in Schleswig–Holstein, one person (2.9%) each in Baden-Wuerttemberg and in Hesse. Four participants (11.8%) reported a direct (P03, P34) or indirect (P10, P36) MB. Thirteen persons (38.2%) reported having children, with the number of children varying between one and three. The SES of the participants was low in six (17.6%), medium in 18 (52.9%) and high in 10 (29.4%). The profiles of the included participants are detailed in Table 1. The profiles of the excluded participants can be found in Appendix 8.

**Table 1:** Profiles of included healthcare workers in the interview study on stigma during the COVID-19 pandemic

Participant	Age (years)	Gender	Occupation(s)	SES	MB	Duration (min)
P01	23	f	MS	m	no	90
P02	52	f	NU	m	no	86
P03	35	f	MS	m	yes^d^	79
P04	25	f	MS, NU	m	no	72
P05	30	f	DO	h	no	90
P06	25	f	MS	m	no	64
P07	44	m	NU	m	no	75
P08	57	f	NU	m	no	89
P09	23	f	MS	m	no	64
P10	26	f	MS	m	yes^i^	86
P11	24	f	PS, NU	m	no	63
P12	37	f	DO	h	no	49
P13	27	m	PS, NU	m	no	65
P14	26	f	MS	m	no	82
P15	24	m	MS	l	no	80
P16	39	f	NU	m	no	78
P17	24	f	MS	l	no	75
P18	57	f	OT	m	no	65
P19	23	f	MS	l	no	64
P20	21	m	OT	l	no	43
P21	26	f	MS, NU	l	no	35
P22	33	f	MS, NU	m	no	83
P23	52	f	NU	h	no	28
P25	27	f	MS, NU	l	no	56
P26	63	m	DO	h	no	85
P28	52	m	DO	h	no	21
P29	39	f	DO	h	no	41
P30	46	m	DO	h	no	24
P31	41	f	DO	h	no	20
P32	33	m	DO	h	no	32
P33	39	f	DO	h	no	75
P34	23	m	OT	m	yes^d^	30
P35	24	m	OT	m	no	65
P36	19	f	MS	m	yes^i^	30

Gender: *f* female, *m* male. Occupation(s): *MS* Medical students, *NU* Nurses, *DO* Doctors, *PS* Psychology students, *OT* Other HCW. *SES* SES Socioeconomic status, *l* low, *m* medium, *h* high, *MB* MB Migration background, *yes*^*d*^ direct MB, *yes*^*i*^ indirect MB

### Intercoder agreement

Eight interviews (23.5%; 8/34) were separately coded by two independent coders and discussed. The chance-corrected coefficient Kappa (κ) for the intercoder agreement of these interviews ranged between κ = 0.65 and κ = 0.75 and is therefore considered substantial [46]. The percentage agreement and the four-field tables as the basis for the calculations of the chance-corrected coefficients Kappa (κ) are reported in Appendix 9.

### HCWs' experiences of stigmatization during COVID-19 aligned with the HSDF

**Fig. 1:**
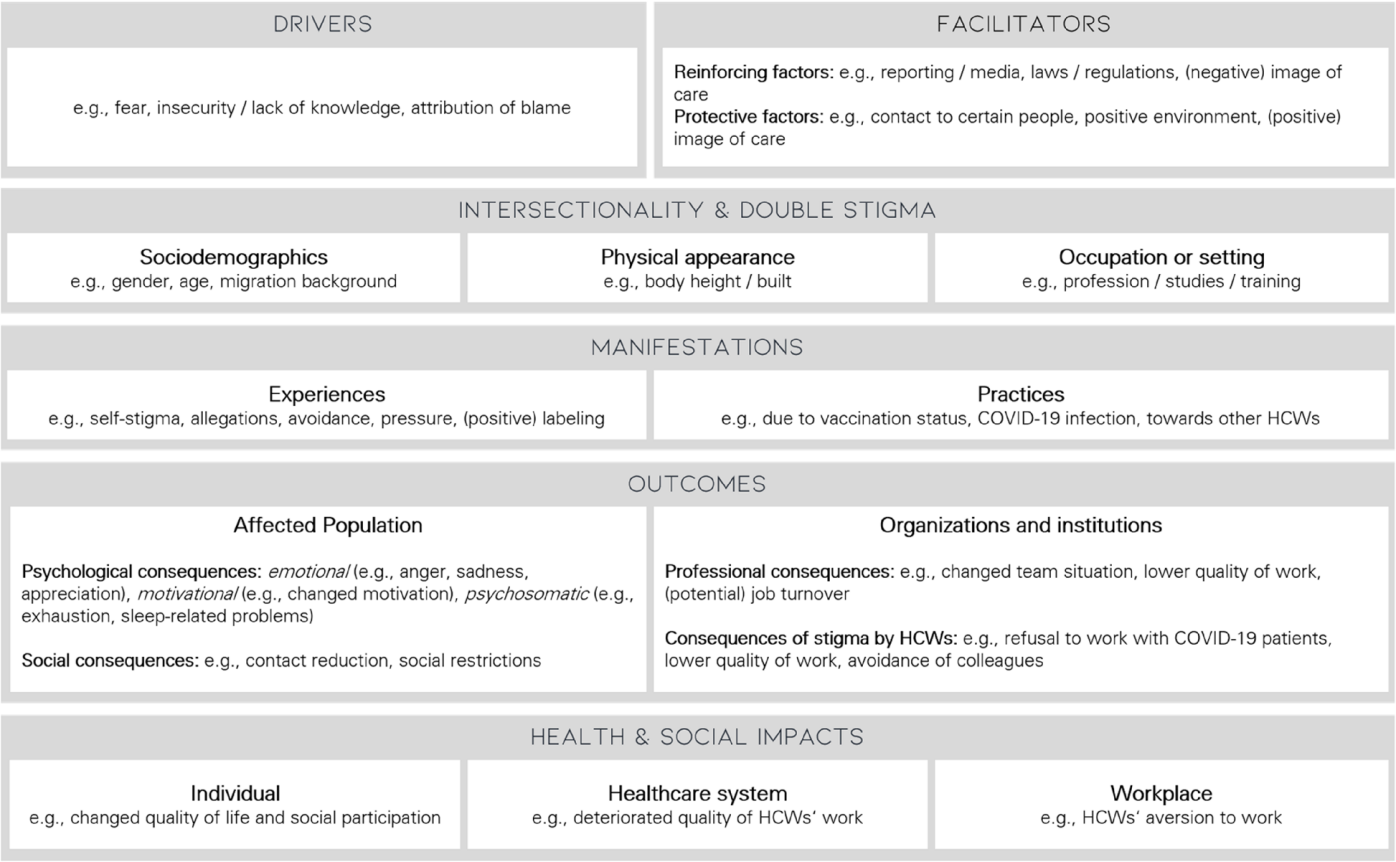
Key findings of healthcare workers’ experiences on stigma during the COVID-19 pandemic

The qualitative findings regarding the content of the main categories, aligned with the HSDF (Fig. 1), provide insights into how HCWs in Germany experienced stigmatization during the COVID-19 pandemic. The key findings for each category are briefly summarized.

#### Drivers and facilitators

The most frequently presumed drivers were individual predictors such as fear of infection and more serious consequences among others, like 'getting sick, dying' (P16), which led to stigmatizing behavior towards HCWs. HCWs also frequently mentioned their own fear of infecting others as a reason for (self-)stigmatization. Stigmatization was often attributed to insecurity or lack of knowledge of others, particularly regarding COVID-19-related information, or (expected) attributions of blame (e.g., regarding infecting others). Other drivers were assumed to be absences from work due to illness, which were for instance, described as 'fatal' (P14), one's own vaccination status or vaccine hesitancy, as well as the vaccine hesitancy of others, who, for example, were afraid of getting vaccinated with 'some kind of poison' (P25). Moreover, some participants reported dissatisfaction or perceived injustice, e.g., regarding financial compensation for health services during the COVID-19 pandemic, as reasons for stigmatization.

Facilitators refer to reinforcing and protective factors that affect the perceived intensity and impact of stigma. For example, most participants who experienced stigma mentioned media coverage and reporting as *reinforcing factors*, describing them as 'sometimes very inflammatory' (P10). Legislation and politics, such as inconsistent regulations, were also mentioned, as well as the general societal value or image of care work, which was described as 'not very high' (P02). In their eyes, stigma was reinforced by existing inadequacies in the healthcare system, conspiracy theories (e.g., about COVID-19 vaccination), and a wide range of opinions about COVID-19 ('both sides. Both, people who talked it down and people who, of course, reacted hypochondriacally to it', P26), excessive demands such as 'too many calls, too few ambulances, too little staff, too many patients' (P35), and the economic orientation of hospitals, as 'commercial enterprises [that] want to keep their profits high' (P33).

On the other hand, some participants indicated that they did not experience any stigmatization in the context of the COVID-19 pandemic while working in healthcare, which provided insight into *protective factors against stigmatization*. For example, HCW’s contact with certain groups of people (e.g., individuals with a medical background or people minimally affected by COVID-19), and a positive environment, especially within the family or workplace were perceived as protective, because they allowed HCWs to discuss and reflect their experiences and counteract (self-)stigma. Furthermore, sufficient education about the illness, the general appreciation of HCWs, responsible behavior of others regarding protective measures, clear regulations at work on how to act during a pandemic situation, and holding a leadership position in healthcare were described as protective factors against stigmatization.

#### Intersectionality and double stigma

In addition to experiencing stigmatization in the context of the COVID-19 pandemic, almost two-thirds of the HCWs reported *other experiences of stigmatization* related to their work. These were most frequently based on their professional roles, especially within the nursing field, and the associated societal assumptions. For instance, participants described perceptions of nurses as the 'go-to girl for everything' (P07) and noted that 'little competence is attributed to nursing' (P21). Stigmatization based on female gender was also frequently mentioned, like misogynistic and sexist comments, disadvantages for their career (such as getting part-time jobs vs. full-time jobs or getting questioned in their decision to work in male-dominated fields like surgery) and doubts about their professional competence, for example not being perceived as (future) doctors and repeated labeling as 'sister' (P31, P12, P09), an outdated, German term referring to nurses that diminishes their professional status. A female medical student, P10, even reported that 'gender is still, unfortunately, the biggest shortcoming of all' and described it as 'the even greater evil' compared to the stigmatization experienced during the COVID-19 pandemic. Moreover, younger participants reported age-based stigmatization, for example, 'not [being] taken seriously and being ridiculed, [while] on the other hand they should be young and energetic, to do the job' (P10) and are perceived to have a 'strong immune system' (P01), implying they would be less affected by the virus.

Less frequently, participants reported experiencing stigmatization related to their MB or origin, e.g., comments about their proficiency in German despite their foreign surname or being questioned about their citizenship, but also related to their physical appearance (e.g., body height, build) or due to the use of naturopathic treatment methods as part of their work. One doctor, for example, described herself as being perceived as an 'herbal witch' (P33), a derogatory, stigmatizing term for those who practice alternative medicine, and stated that she was 'ridiculed' for practicing such methods. Stigmatization based on (female) gender (young) age, and profession, studies, or training often intersected: especially young, female (future) physicians felt perceived as a 'little blonde girl, little blonde nurse […][that] cannot do that much, [and] know[s] that much.' (P04) as well as experiencing sexist comments or even assaults. In contrast, one male paramedic, P20, reported that his age was positively acknowledged, 'to have such a demanding job at such a young age' and stated that 'his competence was never denied,' emphasizing that such intersectional stigma could be a greater problem for female HCWs.

A detailed comparison of the forms of stigma and occurrences of double stigmatization between physicians and nurses can be found in Appendix 10.

#### Manifestations

Approximately three-quarters of the HCWs reported directly *experiencing stigmatization* in the context of their work during the COVID-19 pandemic. This included various forms of stigmatization, with self-stigmatization being reported most often, for example 'My thoughts, when I was directly on that [COVID-19] ward, were, oh man, the main thing is that I don't infect [my mother]. I don't bring the germs home.' (P07, a nurse working in a COVID ward). Other forms of stigmatization included allegations (e.g., due to refusal of the COVID-19 vaccine or working in healthcare during the pandemic), demands for explanations or statements on pandemic-related topics (e.g., because they worked in healthcare or regarding their vaccination status), and pressure or coercion (e.g., regarding the mandatory vaccination for HCWs or avoiding private gatherings). Participants also reported being avoided or excluded (e.g., due to working in a hospital during the pandemic or not being vaccinated), verbal attacks such as being called an 'idiot' for being vaccinated (P15), or conversely, attacks on unvaccinated participants, who were 'very very openly and also very frequently attacked' (P08), and increased inquiries about their vaccination status or work in the hospital. Additionally, some participants described experiences of labeling or exposure, such as a 'small sticker on the name tag at the clinic showing you're vaccinated, like a QR code' (P15). Another form was anticipated stigmatization like being perceived as 'potentially already somehow ill' (P15) due to working in healthcare. Some participants also mentioned positive stigmatization, such as being labeled as 'the group that’s basically still keeping the world in order' (P01) or as 'hero[es]' (P14, P16, P28), although it was noted, that this was limited 'only [to] a short time' (P16).

Common sources of stigmatization were family, acquaintances, neighbors, friends, and flat mates as well as employers, (direct) superiors, colleagues, patients, and their relatives. Participants reported less frequently that they had experienced stigmatization in a public context, i.e., from strangers, the general public, the government, or the media.

In addition to their own experiences, the majority of HCWs also reported *stigmatizing practices* towards others in the context of their work during the COVID-19 pandemic. This comprised stigmatization of vaccinated and unvaccinated persons, and towards individuals who did not comply with protective measures, for example, being 'more cautious towards [other] staff', that they perceived 'a bit careless' (P36) in complying with e.g., wearing a mask. Stigmatization towards persons with a (potential) COVID-19 infection, persons who attended celebrations or festivities, other HCWs, and (by HCWs explicitly labeled) 'vaccination opponents' (P20, P25, P26) were also frequently reported. Less frequently reported forms of stigmata were directed at individuals who did not believe in COVID-19, those with poor health behaviors, persons perceived as having many social contacts, homeless individuals, and those with other nationalities or languages. Six HCWs explicitly reported no stigmatizing behavior or attitudes in this context.

#### Outcomes for affected populations and institutions

Affected HCWs mainly reported psychological and social, but also professional consequences due to their experiences of stigmatization. *Psychological consequences* were expressed in various aversive emotions such as irritability, anger, disappointment, and sadness. In addition, feelings of coercion or restriction, such as not feeling 'completely free' (P15), and feelings of rejection were reported. Occasionally, feelings of unfairness, which were described as 'strongly negative feeling[s]' (P10), frustration, resignation and helplessness, insecurity and responsibility, shame, and loneliness were mentioned by the participants. Less frequently, psychological consequences were also expressed in appetitive emotions such as feelings of appreciation and increased resilience to similar situations. Motivational consequences, such as both, increased and decreased motivation, as well as a stimulated thirst for knowledge concerning COVID-19-related topics were expressed. Psychosomatic consequences, such as exhaustion and tiredness, or sleep-related problems, were also mentioned.

The *social consequences* of stigmatization, i.e. the effects on the persons' family and private lives, were mainly expressed in terms of reduced contact with others and altered social relationships, which were both strengthened and weakened by stigmatization. Social or societal restrictions, such as reduced social participation, with participants limiting their activities to 'only the bare minimum' (P03) were mentioned. A demand for information, as reported by P01 'that [she] was on the front line and was actually also a reporter', whereby the military choice of the word 'front line' as a term for healthcare work was striking here, indicating war-like conditions, were also reported. One participant even reported experiencing resentment towards her, as she was accused of having 'earned a golden nose' (P06) while working during the COVID-19 pandemic, implying that she had made a lot of money with little effort. Another participant P13 explicitly mentioned that there were no social consequences due to stigmatization as the primary contact with persons from a medical background had sufficiently protected him from this.

With regard to the participants' statements on the *professional consequences* of stigmatization, i.e., the effects on their working life, it is apparent that, in most cases, no professional consequences were explicitly reported. However, there were frequent reports of disturbed work processes, more difficult work (and networking) as well as changes in the team situation, including both positive and negative changes. In addition, changes in interaction with patients, like being more cautious or more demanding, lower quality of the HCWs' work, a worse general mood at work, and even consideration of job turnover were further mentioned.

Alongside the stigmatization they experienced, the stigmatization practiced by HCWs themselves has had consequences for their professional practice, for example:

Well, when I think about it, right in April 2020, when I had to go to this corona ward, my stigma, if I can call it that, was relatively blatant. Because it was a new situation, I had been caring for my mother at home at the age of 80 at the time and I had actually resisted [to work], as my mother was a high-risk patient over 80. (P07)

Additionally, some participants reported a deterioration in the quality of their work due to their own stigmatization (e.g. working faster with COVID-19 patients) as well as more strenuous work, tensions within the team (e.g. caused by differing opinions on vaccination), avoidance of colleagues and a more irritable general mood at work.

#### Social and health-related impacts

In addition to the consequences mentioned, the HCWs also reported impacts of their stigmatization experiences on an *individual level*, such as a reduced quality of life, e.g., due to insecurity, external pressure, or a lack of social contacts. The extent of the negative impact ranged from 'hardly at all' (P06) to a 'moderate' (P22) influence, up to a 'significantly reduced quality of life' (P05). Positive impacts on quality of life, such as the perception of being 'tough[er]' (P10) as a result of these experiences, were rarely reported. Two persons explicitly stated that the stigmatization had no impact on their quality of life.

Most participants further stated that their social participation was limited, mainly due to a lack of social meetings. The extent of the negative impact on social participation also ranged from 'actually not that big of an influence' (P01) to a 'medium to large influence' (P33) and up to the 'social factor [being the] greatest limitation' (P22). Less frequently, it was explicitly reported that the stigmatization had no influence on social participation. Only one participant (P10) reported a positive influence on social participation through compensatory behavior.

### Coping strategies

**Fig. 2:**
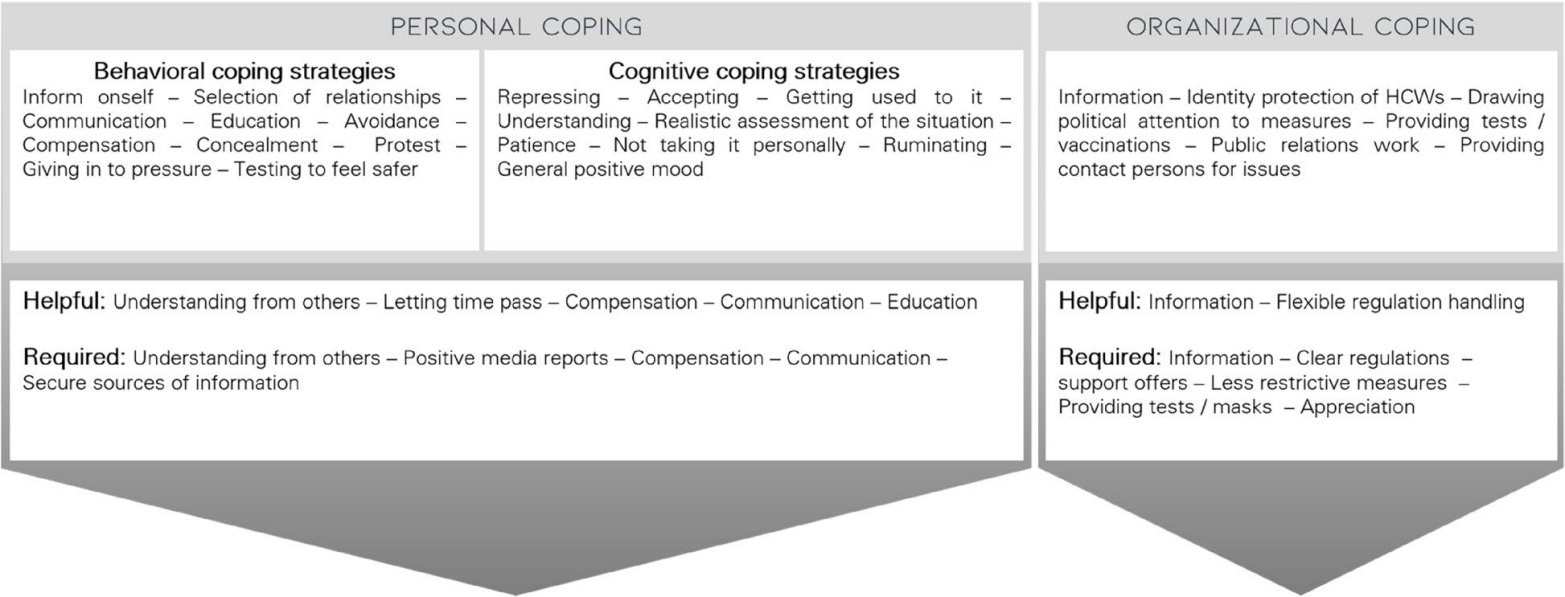
Personal and organizational coping strategies of healthcare workers experiencing stigma during the COVID-19 pandemic

For a visual summary of the coping strategies reported by HCWs, see Fig. 2.

In order to personally cope with the experiences of stigmatization, participants reported employing both behavioral and cognitive coping strategies. *Behavioral coping strategies* were predominant and involved engaging in conversations or sharing stigmatization experiences with others, such as colleagues or family members, which was described as uplifting and a 'mutual reinforce[ment]' (P06) by some but 'a bit exhausting' (P13) by others. Participants stated that they tried to educate and explain their work to people perpetuating stigmatization. Avoidance or distancing behaviors to cope with (self-)stigmatization were also common, e.g., avoiding COVID-19-related conversations, replacing face-to-face contact with online meetings, or avoiding social gathering altogether, as described by one participant: 'avoiding contacts [as a] way of dealing with the fear of somehow becoming infected and/or infecting someone else.' (P14). Some participants avoided the topic by withholding information (e.g., regarding vaccination status or illness symptoms). Participants also carefully selected social relationships, choosing to engage with individuals who shared similar interests or professional roles. Other participants reported that they got vaccinated because of external pressure or took regular COVID-19 tests to increase their feeling of safety. One participant actively sought information about legal regulations, while another reported involvement in 'Anti-COVID-Protests' (P33) as a coping strategy.

*Cognitive coping strategies* comprised understanding with people who displayed stigmatizing behavior, such as recognizing their fear as a plausible reaction. Participants also reported adopting a generally positive mood, maintaining patience with the situation, and making realistic assessments of circumstances in order to cope with their experiences of stigmatization. Other cognitive approaches included reframing stigmatization by not taking it personally and accepting it without getting angry. Some participants coped by processing the stigma through reflective thinking or rumination, while others mitigated its impact by choosing not to worry or actively repressing related thoughts.

The participants explicitly reported that compensation like sport or relaxation, conversations (e.g., with people who have had similar experiences), and understanding from others were both helpful and required in coping with stigmatization. Education of others and oneself and letting time pass were also reported as helpful strategies. Furthermore, the need for secure sources of information regarding COVID-19-related topics and positive media reports (e.g., highlighting successes in healthcare) were emphasized.

Regarding how the organization or employer dealt with the experiences of stigmatization, most participants stated that they had not noticed any measures or they reported the provision of protective measures like tests or vaccinations for HCWs, which can reduce stigma by creating and communicating a sense of safety for both HCWs and others. The provision of COVID-19-related information materials was also reported, as well as public relations work by the organizations and raising political attention to the measures. Less frequently, HCWs were supported through contact persons in case of problems (e.g., a helpline) or the organization's protection of the identity of infected staff to avoid stigmatization.

Some HCWs mentioned the provision of information by experts (especially on COVID-19-related topics) and a more relaxed approach to workplace regulations in the context of the COVID-19 pandemic as helpful organizational forms of dealing with the situation. On behalf of the employers or organizations, some participants stated that they needed offers of help or support, e.g., in the form of communication training, clearly communicated regulations in the work context, and less restrictive actions from employers to better cope with stigmatization. In addition, the provision of tests or masks, information material, and appreciation (e.g., financial and non-material), were mentioned as desirable.

## Discussion

This study's purpose was to explore how HCWs in Germany experienced stigmatization during the COVID-19 pandemic as well as examine their coping strategies, needs, and available support, using the HSDF [15, 16]. Using an exploratory qualitative approach and an intersectional perspective, we aimed to enhance the understanding of how stigmatization manifests and affects HCWs.

### Drivers and facilitators

Fear of infection and its consequences were the predominant driver of enacted stigma by others, while self-stigma among HCWs was primarily driven by fear of infecting others. This aligns with the HSDF [16] as well as findings of Faller et al. [17], who highlighted fear of contagion, lack of knowledge or uncertainties, illness-related staff shortages in healthcare, and vaccine skepticism as factors contributing to stigmatization during the COVID-19 pandemic.

Participants identified the media, political decisions, and the low societal valuation of caregiving professions as key factors facilitating stigmatization. These findings align with the assumptions of Ransing et al. [16] within the HSDF, which highlighted the role of media narratives and legislation in intensifying stigma during the COVID-19 pandemic, as well as preexisting social inequalities, such as the undervaluation of caregiving professions. However, contrary to Ransing et al.'s [16] assertion, this study did not observe any religious, cultural, or supernatural beliefs associated with COVID-19 infections among participants.

Protective factors against stigmatization primarily included contact with specific groups of people, particularly those with a medical background, and a positive family or work environment as opportunities for HCWs to discuss and reflect on their experiences. Additionally, the appreciation and valuation of caregiving professions contributed to resilience against stigma.

### Manifestations

The stigma experienced by HCWs during the COVID-19 pandemic included public stigma, anticipated stigma, and self-stigmatization, originating from diverse sources such as private, public, and work contexts. Similar reports can be found in the international literature: Zolnikov and Furio [23] conclude that first responders (including HCWs) mainly experienced stigmatization by their friends and families. Schubert et al. [47] found communities, workplaces, and social surroundings to be sources of stigmatization towards HCWs during the pandemic.

Forms of stigmata identified in our study were comparable to those from Faller et al.[17]. They include distancing and avoidance, accusations and verbal attacks, increased questioning, and self-stigmatization. Ransing et al. [16] also identified self-stigmatization, concerns about job security, and issues in friendships, which were also evident in our findings. Reports of participants being positively labeled’heroes’ in our study was also a common label mentioned [48]. Makino et al. [49] emphasize the importance of not treating HCWs as a uniform entity that must sacrifice itself to fight against COVID-19, but rather, appropriately support and compensate them for their efforts.

The present study did not identify associative stigmatization of HCWs' families, particularly their children, as found in several studies [16–18, 20].

While many HCWs in our study reported experiencing stigmatization from others, a few also acknowledged that they themselves engaged in stigmatizing behaviors in the context of their work during the COVID-19 pandemic. These instances primarily related to colleagues’ or patients’ infection or vaccination status or non-compliance with protective measures. Yufika et al. [27] also found that approximately 22% of HCWs acknowledged engaging in stigmatization related to COVID-19. In our study, stigmatizing others was reported to have negatively impacted HCWs’ professional practice, leading to a refusal to work, a deterioration in the quality of care, and increased tensions within their team. Accordingly, this can negatively influence the quality of care, particularly through a decreased willingness to provide health services, e.g., to COVID-19 patients [16]. This is consistent with research in other healthcare contexts, such as studies on the stigmatization of HIV-positive individuals, which found that stigmatizing attitudes among HCWs acted as a barrier to patient care [50].

### Intersectionality and double stigma

In addition to the stigmatization experiences related to the COVID-19 pandemic, HCWs reported being stigmatized due to the female gender, their professional status (e.g., nurses compared to medical doctors), and their young age. Especially young women in medical professions reported experiencing multiple forms of stigmatization, which highlights the issue of gender-based stigmatization of women in healthcare [51]. Hennein et al. [51] also identified younger age, being a physician (in training), and heightened racial discrimination as significant predictors of increased gender-based discrimination among women in healthcare. However, these experiences did not intersect with COVID-19-related stigma.

In contrast to previous work, this study did not find associations with stigma due to perceived ethnicity (particularly Asian decent), low SES, and older age with stigmatization during the COVID-19 pandemic [12, 14, 52–54]. This discrepancy can be attributed to the sample composition of this study, which did not include individuals of Asian descent and only few older participants, or those with low SES.

### Outcomes and social and health-related impacts

Participants reported that stigma impacted quality of life and social participation, on psychological (e.g., negative emotions like sadness, anger or shame), social (e.g., reduced contact), and professional levels (e.g., changes in team dynamics), which is in line with existing literature [16, 17, 23, 24, 47, 55, 56].

In a scoping review, Negarandeh et al. [57] summarize similar consequences for HCWs: psychological (e.g., stress, sadness, feelings of guilt, anger), physical (e.g., sleep-disturbances, exhaustion), social and professional (e.g., compassion fatigue, job burnout, negative effects on work performance) as well as economic (e.g., economic losses), which were not explicitly mentioned by HCWs in this study.

### Coping strategies

In terms of personal coping, conversations and exchanges about experiences of stigmatization, understanding from others and compensation like sports or relaxation, were perceived as particularly helpful by HCWs. Additionally, the need for trustworthy sources of information regarding COVID-19-related topics and positive media reports were emphasized. These findings align with Faller et al. [17] and Zolnikov and Furio [23] who highlighted the use of communication, particularly with colleagues, and emphasized the necessity of educating the public with scientifically grounded information about the COVID-19 pandemic as key strategies for dealing with stigmatization.

Regarding how organizations or employers addressed stigmatization experiences, HCWs predominantly reported that no measures to counteract stigmatization were observed. When measures were mentioned, the most helpful organizational approaches included the provision of information and fostering a relaxed approach to workplace behavioral guidelines. Participants expressed a desire for clearly communicated behavioral guidelines by employers, as well as organizational support and assistance, to better cope with stigmatization experiences. In a rapid review, Callus et al. [58] summarized effective techniques of stress reduction for HCWs who care for patients infected with severe SARS, MERS, and COVID-19, highlighting the importance of organizational measures (e.g., clear communication, ensuring safety and well-being, accessible mental health services), tailored interventions (e.g., like digital tools or relaxation resources), and self-care strategies (e.g., mindfulness-based interventions, acting on self-efficacy).

### Strengths and limitations

The face-to-face (online) format of the interviews enabled rich and flexible data collection. However, the potential for response bias, such as social desirability effects, and selection bias due to the online format, cannot be entirely excluded [59, 60]. A further limitation is the potential for recall bias, particularly as most data collection took place after the official end of the COVID-19 pandemic, which could have affected participants’ memories [61]. Since we recruited participants in two waves, more time has passed for participants in the second wave. But since they differed in many other aspects (e.g., mostly men, mostly physicians) from the first wave, it was not possible to fully analyze the impact of recruitment time on their responses.

Another limitation of the study is the demographic imbalance in the sample, with under-representation of male participants, those with MB and those with low SES, a majority being from Mecklenburg-Western Pomerania, and the inclusion of only a few HCWs from other occupations within the healthcare system (e.g., geriatric care), which can restrict the generalizability of the results to the broader population of HCWs in Germany. Nevertheless, the higher representation of women aligns with the gender distribution in healthcare professions in Germany, where approximately 75% of the workforce in healthcare is made up of women [61].

Additionally, the characteristics and assumptions of the researchers can influence the research process and need to be critically reflected (so-called subjective reflexivity) [62]. To counteract subjectivity, several researchers, that varied in gender, age and MB, were involved in the research process.

### Implications for practice and policy

Based on the results of the present study, *practical implications* for support measures and needs related to the stigmatization of HCWs can be derived. An important approach to prevent workplace stigmatization is establishing and communicating behavioral guidelines during infectious disease outbreaks within each organization. This could help mitigate uncertainties, fears, and blame that may lead to (self-)stigmatization. Communicating unified, evidence-based information to the general population and HCWs could further support individuals to assess the situation based on evidence and reduce the spread of misinformation [16, 19]. Epidemiological findings and the reports and experiences of HCWs should be considered in this context [23]. Besides valid information, communication should use suitable terminology (e.g., ‘physical distancing’ instead of ‘social distancing’) and avoid stigmatizing language to prevent further stigmatization [16]. Healthcare organizations should provide accessible psychological support for HCWs, including online services during periods of isolation [63, 64]. Furthermore, implementing anti-stigma trainings for HCWs may help reduce provider-based stigma by offering information and practical skills [65, 66]. *Policymakers* should also recognize that measures or laws targeting specific population groups can contribute to their stigmatization and needs to be considered in policy-making processes. Measures such as providing protective equipment, offering support services, and ensuring compensation through employer initiatives are recommended to support HCWs in coping with stigmatization experiences. It is also crucial for HCWs to receive appropriate recognition, both socially and financially [49]. Given that HCWs report negative feelings and restrictions on their quality of life and participation, which could increase the risk of mental illness, psychological support or access to contact persons at work should be made available [47].

Since HCWs experience stigmatization from various sources, Schubert et al. [47] recommend building on the categorization by Heijnders and van der Meij [67], to implement anti-stigma measures in these different contexts and at various levels, i.e., at the intrapersonal, interpersonal, organizational, structural, or community level. Intersectional aspects should also be considered, such as programs that promote gender equality and thus reduce structural stigmatization [51]. In this context, young women (in medical professions) should be specifically addressed and supported, and interventions should be tailored to specific target groups. Additionally, effective coping strategies should be promoted and reinforced. For example, measures fostering a positive work environment and strengthening team cohesion to counteract workplace stigmatization proactively. Moreover, (online) self-help groups for HCWs could provide a platform for sharing experiences and mutual support to encourage coping with stigmatization [68]. Finally, raising awareness among HCWs about their own stigmatizing behaviors toward others in their work environment is essential, as this can negatively impact their work practice [16].

### Directions for future research

The present study highlights the need for future *research* on stigmatization processes in the context of infectious disease outbreaks, like the COVID-19 pandemic, to adopt an intersectional perspective. The findings indicate that the interplay of personal characteristics can lead to multiple stigmatizations of HCWs in the context of their work. Adopting this perspective would enable researchers to capture these processes' complexity better and identify particularly vulnerable groups [69, 70]. Furthermore, qualitative research should be a foundation for subsequent quantitative studies, that could quantify the degree of stigmatization or its impact on individuals using representative samples. This would make it possible to identify groups particularly affected by stigmatization (e.g., based on gender, profession), consequences that are most burdensome and require greater attention, or particularly effective coping strategies in dealing with stigmatization. This approach could help verify whether the results of the present study can be generalized to HCWs in Germany. Finally, future research should further expand the study by balancing demographic variables of the sample. This includes actively recruiting male participants, individuals with a lower SES, and those with an MB.

## Conclusion

In the context of the COVID-19 pandemic, HCWs faced not only increased workload but also significant stigmatization, which even persists after the pandemic, resulting in a range of mainly negative consequences. Given the crucial role HCWs play in managing infectious disease outbreaks, the importance of addressing their stigmatization in research, policy, and practice becomes evident. The identified needs and effective coping strategies of HCWs need to be recognized to provide optimal support in similar situations. Additionally, the relevance of multiple stigmatizations and various dimensions of disadvantage should be considered to reflect the complexity of stigmatization processes adequately.

## Supplementary Information

Supplementary Material 1.

## Data Availability

All study materials are available as Appendices Open Access at https://osf.io/dpq3k/?view_only=a24535ed2a644cd99c9624480a855637 in German (original version) and English (translation). For an overview of the Appendices, see Supplementary Material. The anonymized data of the current study are available from the corresponding author (DD) on reasonable request.

## Abbreviations

SARS-CoV-2: Severe Acute Respiratory Syndrome Coronavirus-2
COVID-19: Coronavirus disease 2019
HCWs: Healthcare workers
HSDF: Health Stigma and Discrimination Framework
MB: Migration background
SES: Socioeconomic status
