# Supplementary material for: Experiencing stigmatization during the COVID-19 pandemic: a qualitative study among healthcare workers
Source: BMC Infect Dis. 2025 Sep 9;25:1104. doi: 10.1186/s12879-025-11486-1 (PMC12418709; doi:10.1186/s12879-025-11486-1)
Supplement: Supplementary file 1 — Supplementary Material 1. [file 12879_2025_11486_MOESM1_ESM.docx]

# Overview of Appendices (English Version)

Appendix 1 Socio-demographic questionnaire

Appendix 2 Interview guideline

Appendix 3 Interview protocol

Appendix 4 Anonymization rules

Appendix 5 Coding rules

Appendix 6 Codebook

Appendix 7 Final coding system

Appendix 8 Profiles of the excluded participants

Appendix 9 Tables for assessing the intercoder agreement

Appendix 10 Comparison of stigmatization experiences across different professional groups in the healthcare sector

All study materials are available as Appendices in German and English at: <https://osf.io/dpq3k/?view_only=a24535ed2a644cd99c9624480a855637>

# Anhangsverzeichnis (Deutsche Version)

Anhang 1 Soziodemografischer Fragebogen

Anhang 2 Interview-Leitfaden

Anhang 3 Interview-Protokoll

Anhang 4 Anonymisierungsregeln

Anhang 5 Codierungsregeln

Anhang 6 Codebuch

Anhang 7 Codesystem

Anhang 8 Profile der ausgeschlossenen Teilnehmenden

Anhang 9 Tabellen zur Beurteilung der Intercoder-Übereinstimmung

Anhang 10 Vergleich der Berufsgruppen im Gesundheitswesen hinsichtlich ihrer Stigmatisierungserfahrung

Alle Materialien sind auf Deutsch und Englisch verfügbar unter: <https://osf.io/dpq3k/?view_only=a24535ed2a644cd99c9624480a855637>
